# Research progress and treatment status of malignant ascites

**DOI:** 10.3389/fonc.2024.1390426

**Published:** 2024-12-16

**Authors:** Jing He, Hui-ping Zhang

**Affiliations:** ^1^ Department of Traditional Chinese Medicine, The First Affiliated Hospital of Dali University, Dali, China; ^2^ Department of Oncology, Guang’anmen Hospital Jinan Hospital (Jinan Hospital of Traditional Chinese Medicine), Jinan, China

**Keywords:** malignant ascites, treatment, research progress, malignant tumors, oncology

## Abstract

Malignant ascites (MA), a common and serious complication of various cancers in the abdominal cavity, originates from the extensive infiltration, metastasis, and growth of cancer cells in or on the abdominal cavity, leading to abnormal accumulation of fluid in the abdominal cavity and the formation of MA. MA seriously reduces the quality of life of cancer patients, shortens their survival period, and generally has a poor prognosis. Modern medicine has developed various strategies for the treatment of MA, including targeted supportive treatment, diuretic treatment, abdominal paracentesis, surgical intervention, and intraperitoneal administration therapy. Among them, chemotherapy, as one of the important treatment methods, includes both systemic chemotherapy and intraperitoneal chemotherapy, especially pressurized intraperitoneal aerosol chemotherapy (PIPAC), hyperthermic intraperitoneal chemotherapy (HIPEC), and foam-based intraperitoneal chemotherapy (FBIC), providing a new choice for the treatment of MA. In addition, innovative treatment methods such as gas-based intra-abdominal hyperthermia (GIH) combined with dehydration therapy have also shown promising application prospects. This article delves into multiple aspects of MA, including its concept, mechanism of occurrence, clinical manifestations, differential diagnostic methods, and current treatment status and research progress. This comprehensive review aims to provide valuable references for effectively controlling MA, improving cancer patients’ quality of life, and prolonging the survival cycle of cancer patients in clinical practice. Malignant ascites (MA) is a common complication of cancer, which originates from the extensive infiltration, metastasis, and growth of cancer cells in the abdominal cavity or peritoneum, leading to abnormal accumulation of peritoneal fluid. It is a common clinical manifestation in the late stage of cancer. Its symptoms are stubborn and recurrent, which can lead to abdominal pain, bloating, poor appetite, fatigue, breathing difficulties, and even multiple organ failure. The median survival time for cancer patients with MA is generally 5 to 6 months. The prognosis is poor, and it is imperative to seek more active and effective treatment plans. This article reviews the research and treatment status of MA, aiming to provide certain value for controlling MA and improving the quality of life of patients.

## 1 Concept

The abdominal cavity is the largest in the human body, which is a potential serosal cavity formed by the mutual extension of the visceral peritoneum and the parietal peritoneum (1). Under normal conditions, a small amount of physiological peritoneal fluid (usually less than 200 mL) (2) is present in the abdominal cavity of the human body. This fluid comes from the plasma filtered out by the capillaries in the abdominal wall and is recovered through the lymphatic vessels and small veins in the visceral peritoneum. It lubricates the visceral, parietal peritoneum, and intestinal peristalsis, appearing colorless, transparent, or light yellow. When any pathological condition leads to more than 200 mL of fluid retention in the abdominal cavity, it is called ascites. According to the causes, properties, and characteristics of ascites, it can be divided into leaky, exudative, and sanguinous ascites. Generally, MA is exudative, and mixing the three is common. When blood is mixed, sanguineous ascites is formed.

## 2 Production mechanism

The physiological and pathological mechanism of MA formation is very complex and has not been fully elucidated. It is generally believed to be influenced by multiple factors, among which lymphatic vessel obstruction and changes in vascular permeability are the most critical factors.

### 2.1 Obstruction of lymph and tissue fluid return

Under physiological conditions, peritoneal fluid is reabsorbed through the subperitoneal lymphatic vessels, mainly in the lymphatic capillaries under the diaphragm (3). When cancer cells continue to increase, obstructing the lymphatic vessels under the diaphragm, the return of lymph and tissue fluid will be blocked, resulting in fluid retention in the abdominal cavity. In addition, direct compression of tumor or lymph node enlargement, as well as obstruction of the portal vein, hepatic vein, and inferior vena cava reflux caused by cancer thrombus formation, can also lead to reduced tissue fluid reflux and leakage into the abdominal cavity.

### 2.2 Neovascularization and increased vascular permeability

The infiltration of tumor cells into the peritoneum or intestinal wall, as well as inflammatory stimulation of cancer, may promote endothelial damage and increase vascular permeability. During the growth and development of tumors, tumor cells can secrete or induce normal cells to secrete related influencing biological factors, such as vascular endothelial growth factor (VEGF) (4), interleukin-6 (IL-6) (5), matrix metalloproteinases (MMPs) (6), tumor necrosis factor (TNF) (7), vascular permeability factor (VPF) (8), and cell adhesion molecules (CAMs) (9). These factors can induce tumor neovascularization, increase the number of new blood vessels, and increase the expression of tumor cell glycoproteins. Under the combined influence, vascular permeability increases, leading to an increase in protein concentration in the abdominal cavity, as well as to an increase in colloid osmotic pressure in the abdominal cavity, ultimately forming MA (10).

### 2.3 Increased permeability of microvascular walls

Under normal circumstances, only trace proteins are allowed to filter out from the capillary walls, resulting in a large colloidal osmotic pressure gradient inside and outside the capillaries. When the tumor metastasizes to the adjacent peritoneum and omentum or is widely implanted and metastasized in the abdominal cavity, it may cause an increase in local peritoneal capillary permeability (11), and a large amount of plasma albumin may extravasate into the tissue gap, causing fluid to accumulate in the tissue gap and form MA.

### 2.4 Reduced plasma colloid osmotic pressure

As the cancer continues to develop, there will be sustained nutrient depletion in the patient’s body. At the same time, liver dysfunction will weaken the liver’s ability to synthesize proteins (12). When the above situation leads to a decrease in albumin concentration in the plasma below 30 g/L, it will trigger hypoalbuminemia. One important consequence of hypoalbuminemia is the decrease in plasma colloid osmotic pressure, which weakens the ability of plasma to maintain normal osmotic pressure (13). Water in the capillaries infiltrates into tissue gaps, leading to the formation of ascites in the abdominal cavity.

### 2.5 Activation of relevant regulatory mechanisms

On the one hand, the large number of MA produced can lead to the formation of new compartments in the body that store extracellular fluid, causing a decrease in effective circulating blood volume. On the other hand, cancer patients may experience a reduction in blood volume or an insufficient supply of blood to various tissues and organs due to blockage or poor circulation. A decrease in intravascular volume activates the renin–angiotensin–aldosterone (RAAS) (14), reduces glomerular filtration rate, and stimulates the secretion and enhancement of vasoactive substances such as atrial natriuretic peptide, prostaglandins, and antidiuretic hormone, to maintain stable blood pressure. These mechanisms will lead to increased water reabsorption, decreased urine output, and retention of water and sodium, thus further increasing the accumulation of MA (15).

### 2.6 Abdominal fluid can be absorbed through the lymphatic vessels and right thoracic duct and cleared after entering the right subclavian vein

When lesions such as retroperitoneal tumors and mediastinal tumors obstruct the thoracic duct or chylous cistern, or when chyle fluid leaks into the abdominal cavity due to traumatic rupture, it can lead to the formation of chylous MA (16).

## 3 Clinical characteristics

MA accounts for approximately 10% of all ascites cases. It is most commonly seen in patients with ovarian cancer [25%–28% of all MA cases (17)], which is one of the reasons why MA is more common in women than men (67% vs. 33%) (18). The next most common primary cancers include cancer from the digestive tract, such as pancreatic cancer, gastric cancer, and colorectal cancer, as well as intra-abdominal tumors, such as primary peritoneal cancer, and extra-abdominal tumors such as lymphoma, lung cancer, breast cancer (19). Additionally, approximately 20% of patients with MA do not exhibit easily identifiable primary tumors (20). Compared to ovarian cancer, patients with primary gastrointestinal tumors and those with unknown primary tumors usually have a poorer prognosis. The median survival period of MA is 5.7 months (21), while the expected life of refractory MA is 1–4 months (22). More than 95% of MA patients have evaluable or measurable metastatic diseases, indicating that the appearance of MA indicates that the patient’s condition is in the final stage and the prognosis is poor.

MA has a serious impact on the quality of life and survival of cancer patients, with clinical manifestations including severe gastrointestinal dysfunction (23), such as nausea, vomiting, loss of appetite, fatigue, weight loss, bloating, abdominal pain, and decreased tolerance to daily activities. Patients with advanced malignancies may experience chest tightness, wheezing, and difficulty breathing. Physical examination may reveal abdominal swelling, increased abdominal circumference, weight gain, positive abdominal mobility dullness and wave sensation, lower limb edema, palpable lumps, tenderness, and rebound pain in the abdomen. Laboratory indicators usually show a decrease in indicators such as albumin and hemoglobin, which can cause the following outcomes: 1) fluid loss and electrolyte disorder: reduced effective blood circulation and a large amount of potassium, sodium, calcium ions, etc. infiltrating the abdominal cavity, leading to electrolyte disorders in patients. 2) Reduced intestinal function: Immersion of the intestinal tract in a large amount of MA may cause a decrease in intestinal peristalsis and digestion and absorption capacity, exacerbating bloating and malnutrition. 3) Abdominal infection: A large amount of MA may cause the displacement of intestinal flora, induce primary peritonitis, and lead to abdominal infection.

## 4 Diagnosis

### 4.1 Laboratory diagnostic methods

#### 4.1.1 Routine examination of ascites

Routine examination of ascites includes testing for color, transparency, cell count, and specific gravity. 1) Color: MA usually appears yellow or bloody, mostly yellow, due to the presence of bilirubin or components of bile. Cancer cells invading the liver or biliary system may lead to bile leakage or accumulation, resulting in yellow ascites. In some cases, MA may also appear bloody, which may be due to cancer cells invading blood vessels in the abdominal cavity, causing blood to leak into the ascites; bloody ascites may also be related to vascular rupture and bleeding caused by malignant tumor infiltration. 2) Specific gravity: The specific gravity of MA is often greater than 1.018. However, it cannot be distinguished from infectious ascites, and approximately 40% of MA has a specific gravity <1.016. 3) Cell count: The number of red blood cells in MA usually exceeds 100,000/μL. The ratio of red blood cells to white blood cells usually exceeds 10:1. When this occurs, the first thing to suspect is MA caused by liver cancer rupture, peritoneal metastasis, or other cancer. However, the sensitivity and specificity of these indicators are low (24) and are influenced by various subjective and objective interference factors.

#### 4.1.2 Biochemical examination of ascites

The biochemical examination of ascites includes the detection of indicators such as protein, ferritin, serum ascitic albumin gradient, fibronectin, lactate dehydrogenase concentration, glucose, ascitic pH, cholesterol, lysozyme, and adenosine deaminase. 1) Protein: MA usually contains high levels of protein, often above 30g/L, and the ratio of ascites to serum albumin is mostly greater than 0.5. 2) Ferritin: Exudative ascites, such as >500 μg/L or ferritin ascites/serum ratio >1.0, often indicates MA. 3) Serum ascites albumin gradient (SAAG): The gradient of MA is usually less than 1.1 g/dL (25), and the portal hypertension secondary to liver metastasis may cause SAAG to be greater than 1.1 g/dL (26). 4) Fibronectin (FN): It is a high molecular weight glycoprotein that exists in the extracellular space of human tissues. FN has a high concentration in MA, often >75 mL/L. 5) Lactate dehydrogenase (LDH): LDH >3.34 in exudative ascites μmol (L s), ascites/serum LDH ratio >0.6, often indicates cancer or infection. If LDH >8.35 μmol (L s) and if the ratio of ascites to serum is greater than 1.0, it highly indicates MA (27). 6) Glucose: Due to the consumption of cancer cells, MA may present low concentrations of glucose levels, often <2.8 mmol/L (28). 7) Cholesterol (TC): Among the lipids in ascites, the determination of cholesterol (TC) is the most valuable for distinguishing between benign and MA. When the TC in ascites is greater than 1.24 mmol/L, it strongly suggests the possibility of cancer (29). 8) Lysozyme: Cancer cells do not contain lysosomes and do not produce lysozyme. Therefore, when the level of lysozyme in exudative or inflammatory ascites does not increase (below 23 mg/L) (30), this usually indicates MA. 9) Adenosine deaminase (ADA): It plays an important role in activating and promoting the differentiation and proliferation of lymphocytes to enhance the body’s immune function. Studies have found that (30) ADA levels in ascites of cancer patients are significantly reduced. The area below the ROC curve (AUC) for ADA diagnosis of benign and MA is 0.797 (30), with good accuracy.

#### 4.1.3 Ascites cytological examination

At present, the gold standard for diagnosing MA in clinical practice is still to search for shed cancer cells in the ascites, and collecting 500 mL of ascites is sufficient for cytological evaluation. However, due to the atypical cell morphology and the influence of sample sampling techniques, although this method has high specificity, its sensitivity is only 50%–60%, and the true positive rate is only 30%–50% (31), with a high false negative rate.

#### 4.1.4 Tumor marker examination

Tumor markers (TMs) are a type of substance secreted and released by tumor cells or their related tissues, which can reflect the presence and growth of tumors and help identify and indicate the nature of tumors. The common TMs currently used for diagnosing benign and MA include carcinoembryonic antigen (CEA), alpha-fetoprotein (AFP), carbohydrate antigen 125 (CA125), carbohydrate antigen 19-9 (CA19-9), etc. CEA is a broad-spectrum tumor marker when its level is >15 μg/L (32), which prompts MA. In ascites caused by primary liver cancer metastasis, AFP levels are commonly elevated (33); CA19-9 is highly correlated with pancreatic cancer, gallbladder cancer, colorectal cancer, and gastric cancer (32). In ovarian cancer, pancreatic cancer, lung cancer, or breast cancer, the level of CA125 is often increased (34). However, the sensitivity of AFP, CEA, and CA19-9 to the diagnosis of MA is 28.5%, 38.0%, and 19.0%, respectively (35). Therefore, their application in clinical diagnosis is limited. The study of Liu et al. (36) showed that TM in MA has better diagnostic performance than in serum, and the combination of TM and cytological examination increased the diagnostic rate of the latter by 37%. Therefore, in clinical practice, joint testing is usually performed, and when combined testing is performed (36), although there is no significant difference in sensitivity, specificity can be significantly improved.

### 4.2 Physical examination

Physical examination is simple and effective in diagnosing ascites, but it is usually difficult to detect when the fluid volume is small (<500 mL). Cancer-induced ascites often accompanies symptoms such as bloating, abdominal pain, nausea, vomiting, loss of appetite, weight loss, and fatigue. At the same time, a lump can be palpable in the abdomen. When the amount of MA reaches 500 mL during abdominal percussion, the percussion in the elbow–knee position method can be used for confirmation. When the result of shifting dullness is positive, it indicates that the amount of ascites is greater than 1,000 mL. The patient’s flanks show obvious bulges on both sides of the abdomen, resembling a frog’s belly. The fluid thrill is positive, indicating a large amount of fluid accumulation in the abdominal cavity, and the amount of ascites is usually greater than 3,000 mL (37). Generally speaking, the most sensitive physical indicator for ascites is mobility dullness, while the most specific is obvious liquid waves. However, the positive predictive values for both tests are very low, with an overall accuracy of only 58%, and positive results may only be produced in the presence of a large amount of ascites (38). Therefore, observing a small amount of ascites through ultrasound is recommended (39).

### 4.3 Imaging examination

#### 4.3.1 X-ray examination

X-ray plain films can be used to assist in the diagnosis of ascites. Pertinent positive findings include the following (35): 1) hepatic angle sign (the absence of the lower edge of the right lateral hepatic angle), 2) medial displacement of the lateral edge of the liver, 3) pelvic fluid, 4) intestinal loops in an aggregated state, 5) intestinal loops in a separated state, and 6) a blurry abdomen throughout the body. However, similar to physical examination, X-ray examination generally produces positive results only in the presence of a large amount of ascites and usually has low sensitivity and specificity.

#### 4.3.2 CT and MRI examination

1) CT attenuation value of ascites (40): MA is generally an exudate, containing more protein, and the CT value of ascites density is often greater than 20 Hu. 2) Peritoneal structure (41): The peritoneum is often nodular or irregularly thickened. The density of the mesentery and greater omentum increases, appearing as a cake-shaped or patchy thickening, with significant enhancement after being enhanced. 3) Intestinal bondage sign (41): It shows aggregation and constriction, often due to adhesion caused by lesion metastasis. 4) Omental sac ascites (42): The ascites is located in the large and small omental sacs, and there is a sign of omental sac effusion. 5) Ascites ADC value (43): MA is rich in protein and chylomicrons and has a high viscosity, so the ADC value is usually lower than that of benign ascites. 6) T1- and T2-weighted images show medium to high signal intensity. CT and MRI can not only directly detect ascites but also help locate the primary lesion; clarify the presence of enlarged lymph nodes and abdominal and pelvic masses, as well as the condition of abdominal organs such as the liver and spleen (44); and make a comprehensive evaluation.

#### 4.3.3 B-ultrasound examination

Ultrasound is of great significance for the detection and quantification of body cavity effusion, with the advantages of repeatability, non-invasiveness, and dynamic observation (45). It is currently the most sensitive imaging diagnostic method for body cavity effusion. Ultrasound can detect a very small amount of fluid accumulation in the body cavity. Generally, there is approximately 100 mL of fluid in the abdominal cavity that can be detected (46). MA can be seen with densely distributed large punctate echoes, some of which can be fragmented (diameter > 3 mm) (47).

### 4.4 Molecular biology examination

Detection of transforming growth factor-beta 1 (TGF-β1) is conducted, and the expression levels of VEGF and MMPs, as well as the detection of telomerase activity in ascites, are important auxiliary diagnostic methods for MA.

#### 4.4.1 Vascular endothelial growth factor

VEGF is a key proangiogenic cytokine that promotes uncontrolled tumor growth and participates in the production of MA. Abdel Razik et al. (48) conducted a prospective study on 315 patients with benign MA, using enzyme-linked immunosorbent assay to evaluate the levels of VEGF in serum and ascites. They found that the VEGF levels in the MA group were significantly higher than those in the benign ascites group, with detection sensitivity and specificity of 94.9% and 89.5%, respectively.

#### 4.4.2 Matrix metalloproteinases

MMPs are secreted by tumor cells, which break down the tissue matrix during tumor spread, destroy the basement membrane structure, and increase vascular permeability, promoting MA growth (49). Zhang Yanli et al. (50) retrospectively analyzed ascites samples from 59 hospitalized patients. They found that the MMP-3 expression level in the MA group (195.75 + 46.81 ng/mL) was higher than that in the benign ascites group (93.87 + 20.65 ng/mL), with *P* < 0.01.

#### 4.4.3 Telomerase

Telomerase is an enzyme responsible for the elongation of telomeres in cells, which activates and promotes the division and proliferation of tumor cells (51). Park Es et al. (52) studied 19 patients with gastrointestinal origin MA and found that the positive rate of detecting telomerase activity was significantly higher than that of benign ascites. The sensitivity range of telomerase activity in MA was 78%–84%, which was higher than that of ascites cytology. Therefore, it can be used as a useful auxiliary diagnostic method.

#### 4.4.4 Transforming growth factor-beta 1

TGF-β1 belongs to the small molecule peptide class and is a group of bioactive substances with cellular regulatory functions, which are highly expressed in tumors (53). In clinical practice, there are certain difficulties in distinguishing between ascites caused by tuberculous peritonitis and MA. He Kun et al. (54) found TGF-β1 in the ascite group of liver cancer patients by grouping and testing 82 patients with MA. The expression level was significantly higher than that of the group with tuberculous peritonitis, indicating TGF-β. The differential diagnosis of the two has a certain value.

### 4.5 Laparoscopic examination combined with peritoneal biopsy

Laparoscopy can visually observe the situation in the abdominal cavity. If a tumor and fluid accumulation are found in the abdominal cavity, it indicates MA. The pathological specimens obtained from peritoneal biopsy have a certain value in determining the source of tumors that cause ascites. The sensitivity of laparoscopic examination combined with peritoneal biopsy diagnosis is as high as 80% (55). However, laparoscopic examination and peritoneal biopsy are invasive surgeries with high risks (35) of organ bleeding, tumor development, and metastasis, and MA patients should use them with caution. On the other hand, patients are often less receptive to invasive procedures due to factors such as physical weakness.

Traditional methods such as routine examination of MA, biochemical examination, cytological examination, and physical examination have relatively limited sensitivity and specificity in distinguishing benign and malignant ascites, and the positive predictive rate is relatively low. Therefore, further imaging examinations, especially ultrasound examinations and tumor marker detection, are needed. In addition, auxiliary methods such as laparoscopic examination combined with peritoneal biopsy and molecular biology examination can be utilized to improve the accuracy and reliability of detection and diagnosis. A single detection method is not scientific enough, and multiple examination methods should be comprehensively adopted. At the same time, it is also necessary to consider the overall situation and risk factors and choose the most suitable diagnostic method. The diagnostic methods and key features of MA mentioned above are summarized in **Table 1**.

**Table 1:** Diagnostic methods and key features of malignant ascites.

Routine examination of MA (24)	Color: yellow or bloody	
	Specific gravity: >1.018	
	Cell count: RBC > 100,000/μL RBC/WBC > 10:1	
Biochemical examination of MA	Protein: >30 g/L	
	Ferritin: >500 μg/L	
	SAAG (25): <1.1 g/dL	
	FN: >75 mL/L	
	LDH (27): >3.34 μmol (L s) and ascites:serum >0.6 >8.35 μmol (L s) and ascites:serum >1.0	
	Glucose (28): low concentration	
	TC (29): >1.24 mmol/L	
	Lysozyme (30): <23 mg/L	
	ADA (30): significantly lower and AUC is 0.797	
Ascites cytological examination (31)	The gold standard for diagnosing MA	
Tumor marker	CEA: >15 μg/L (32)	
	AFP: elevation is common in liver cancer (33)	
	CA19-9: elevation is common in pancreatic, gallbladder, colorectal, and gastric cancers (32)	
	CA125: elevation is common in ovarian, pancreatic, lung, or breast cancer (34)	
Physical examination (35)	Clinical symptoms	
	Abdominal palpation (37)	
	Percussion	The percussion in the elbow–knee position method
		Fluid thrill
		Shifting dullness
Imaging examination	X-ray (35)	Positive results with a high volume of ascites
	CT and MRI (39)	CT attenuation value of ascites (40)
		Peritoneal structure (41)
		Intestinal duct bondage sign (41)
		Omental sac ascites (42)
		Ascites ADC value (43)
		T1- and T2-weighted images: show medium to high signal intensity (44)
	B-ultrasound	The most sensitive imaging diagnostic method (45–47)
Molecular biology examination	VEGF (48)	They are significantly higher in the MA group than in the benign ascites group
	MMPs (49, 50)	
	Telomerase (51, 52)	
	TGF-β1 (53, 54)	
Laparoscopic examination combined with peritoneal biopsy (55)	It can improve the accuracy and reliability of detection and diagnosis	

## 5 Treatment

Handling MA is a significant cross-disciplinary challenge requiring collaborative treatment from multiple disciplines. At present, there is a lack of recognized management and evidence-based guidelines, as well as randomized controlled trials for the best treatment methods (56). The selection and practice of treatment often rely on the experience and professional judgment of clinical doctors. The most basic treatment methods in modern medicine are the use of diuretics and therapeutic puncture procedures (57). The treatment goal is to provide lasting relief of discomfort related to MA, provide palliative treatment, and improve the patient’s quality of life.

### 5.1 Targeted supportive treatment

MA patients are usually in the late stage of the tumor, often accompanied by clinical manifestations such as hypoalbuminemia, malnutrition, insufficient effective circulating blood volume, electrolyte disorders, and infections. At this time, targeted supportive treatment should be emphasized (58). For patients with a small amount of ascites, appropriate exercise is beneficial, but excessive fatigue should be avoided. For patients with moderate and severe ascites, more bed rest can reduce the consumption of heat and protein, reduce the burden of liver metabolism, increase liver and kidney blood flow, and reduce water and sodium retention (59). In addition, it is important to strictly limit sodium intake; adhere to a low-salt, low-fat, and high-protein diet; and supplement sufficient calories and nutrients (60). When needed, intravenous nutritional support and infusion of human serum albumin can improve overall nutritional status (10). In addition, it is necessary to promptly correct electrolyte disorders, limit sodium, and supplement potassium. For anemia patients, blood transfusion treatment should be considered when necessary. Meanwhile, actively controlling infection is also crucial, and appropriate antibiotics need to be selected for treatment based on the severity of the infection (56).

### 5.2 Diuretic treatment

Diuretics have traditionally been used as first-line drugs for treating ascites, with advantages such as simplicity, non-invasiveness, and cost-effectiveness (61). It can reduce ascite volume and alleviate edema. Spironolactone is often used and can be combined with furosemide (62). If the effect is not satisfactory, liver function needs to be rechecked. For patients with moderate to high levels of MA, especially those with liver damage, spironolactone is preferred and may be supplemented with furosemide or hydrochlorothiazide (63). If there is hypoproteinemia (plasma albumin is less than 30 g/L), human albumin infusion can be considered to correct the albumin level (64). At the same time, when using diuretics, it is important to closely monitor the patient’s electrolyte balance and urea levels to avoid electrolyte imbalances. To alleviate the accompanying fluid retention symptoms, furosemide intravenous injection or tolasemide intravenous injection can be considered. The adverse reactions of diuretics include low blood volume, renal failure, nausea, and vomiting. It is worth noting that although diuretics are widely used in the treatment of ascites, their efficacy in treating MA is often unsatisfactory. Multiple previous studies have indicated that diuretics have limited or even no therapeutic effect on some MA patients (65). The overall effective rate is roughly maintained at approximately 40% (66). A study by Lee et al. (67) reported that 61% of MA patients received diuretic treatment, but only 45% of patients observed positive effects. However, for MA patients with secondary liver metastasis accompanied by portal hypertension, the therapeutic effect of diuretics is relatively more positive (68).

### 5.3 Abdominal paracentesis

Abdominal paracentesis (69) is generally performed under ultrasound guidance. This medical procedure involves inserting a puncture needle or catheter into the abdominal cavity to extract a fluid sample or eliminate excess fluid. It is commonly used to diagnose the cause of ascites and relieve abdominal distension symptoms. Although abdominal paracentesis is a relatively safe medical procedure, there are still certain risks, such as bleeding, infection, and organ damage.

#### 5.3.1 Abdominal puncture drainage

For patients with ineffective diuretic treatment, abdominal puncture drainage is the most commonly used method for treating MA symptoms. A physician practice survey (70) reported that 98% of doctors have used puncture surgery, and 89% of doctors believe it is effective. A total of 90% of patients experience rapid but brief relief of symptoms such as bloating, abdominal pain, and difficulty breathing. However, the effect is often short-lived, and some patients may even experience symptom recurrence within 72 h (20). Therefore, in order to control symptoms, patients often need to undergo frequent repeated punctures with an average interval of 10.4 days (56). Becker et al. (1) conducted a multicenter prospective observational study on MA patients admitted to palliative care units, and the results showed that 81% of patients who received puncture treatment, as evaluated by the Numerical Rating Scale (NRS), experienced significant improvement in abdominal distension symptoms. It is worth noting that more than half of patients had to undergo two or more puncture procedures during hospitalization. Repeated puncture and drainage of MA can be accompanied by a series of risks and complications, including (71) decreased effective circulating blood volume, hypotension, pain and even perforation during the puncture process, secondary peritonitis, electrolyte imbalance such as hyponatremia, renal dysfunction, and fluid balance problems caused by hypoalbuminemia, as well as serious complications such as infection and bleeding. Over time, the efficacy of the puncture procedure may gradually weaken, while the risk of complications continues to increase, bringing additional physical and psychological burdens to patients.

#### 5.3.2 Indwelling peritoneal catheter for abdominal drainage

When abdominal puncture drainage leads to serious electrolyte disorders and other complications, or when it is impossible to control fluid increase through continuous puncture, an indwelling peritoneal catheter for abdominal drainage is usually considered. One potential advantage of indwelling catheters is that they can also be used for intravenous infusion, chemotherapy, or targeted therapy. Multiple indwelling catheters have been used for MA management, including non-tunnel and tunnel pleural catheters, tunnel Tenckhoff catheters, non-tunnel Foley self-retaining catheters, scope-type circulating drainage catheters, tunnel and non-tunnel peritoneal catheters, and tunnel peritoneal ports. However, the service life of drainage tubes is limited by functional failures and infections, and catheterization complications include sepsis, cellulitis at the catheter site, leakage at the catheter site, catheter occlusion, and fatal hypotension (68). Due to the high incidence of non-tunnel catheter-related infections and peritonitis, it is usually only recommended for patients with short life expectancy patients with short life expectancy).

### 5.4 Chemotherapy

#### 5.4.1 Systemic chemotherapy

For primary cancer, selecting sensitive chemotherapy drugs can control the tumor and reduce MA. For ovarian cancer, breast cancer, lymphoma, and other cancers that are more sensitive to chemotherapy, systematic treatment is generally used, which may achieve good results. The main reference for the regimen is the systemic chemotherapy guidelines. For example, for ovarian cancer (72), the CAP (cyclophosphamide, adriamycin, cisplatin) regimen or paclitaxel combined with carboplatin can be used; the CHOP (cyclophosphamide, vincristine, adriamycin, prednisone) regimen is chosen for lymphoma (73). For breast cancer (74), CAF (cyclophosphamide, adriamycin, 5-fluorouracil) or combination chemotherapy containing taxus was used. When patients experience moderate to large amounts of MA accompanied by symptoms such as bloating, oliguria, and poor appetite, it is necessary first to alleviate the related symptoms caused by ascites and then undergo systematic chemotherapy after the general situation improves. However, patients with advanced cancer often struggle to tolerate systemic chemotherapy due to physical weakness. According to a research report (12), for cancer patients with MA who cannot undergo tumor resection, the 5-year survival rate after receiving systemic chemotherapy does not exceed 10%.

#### 5.4.2 Intraperitoneal chemotherapy

Intraperitoneal chemotherapy refers to injecting chemotherapy drugs into the abdominal cavity. This method can increase the concentration of drugs in the local area of the abdominal cavity and improve drug permeability and cellular uptake, thereby enhancing the direct cytotoxicity of chemotherapy drugs. The peritoneal barrier and liver first-pass effect limit the entry of chemotherapy drugs into the systemic circulation, mainly through the slow absorption of drugs in the portal vein. This method can reduce the drug concentration in other parts of the body, especially in the bone marrow. The systemic toxic side effects of intraperitoneal chemotherapy are lighter compared to other treatment methods (75).

##### 5.4.2.1 General intraperitoneal chemotherapy

Similar to the guiding principles of systemic antitumor therapy, the selection of intraperitoneal chemotherapy drugs also needs to comprehensively consider the tumor type, patient’s performance status, chemotherapy experience, and potential toxic effects of the drugs. The most commonly used intra-abdominal chemotherapy drugs include paclitaxel drugs, such as paclitaxel and docetaxel; platinum drugs, such as cisplatin and carboplatin; topoisomerase inhibitors, such as irinotecan; and antimetabolic drugs, such as 5-fluorouracil (5-FU) (76). Before using drugs such as fluorouracil, paclitaxel, mitomycin, and epirubicin, the risk of causing peritoneal fibrosis and connective tissue damage should be carefully evaluated.

##### 5.4.2.2 Pressurized intraperitoneal aerosol chemotherapy (PIPAC)

It is a treatment method guided by laparoscopy that increases intra-abdominal pressure by inserting a pressure tube with a micropump. This method can directly deliver room-temperature chemotherapy solution into the abdominal cavity in aerosol form. Currently, PIPAC is considered one of the soothing treatment methods for patients with unresectable intra-abdominal tumors. Its advantages include the following (77): no need for major surgery, which helps to reduce the weak cancer patients’ physical burden and avoid potential risks associated with surgery; the dosage of chemotherapy drugs is relatively small and the toxic side effects are few, so they are safe and well tolerated; uniform and widespread distribution of the drugs within the abdominal cavity is ensured; intra-abdominal pressure is increased to counteract interstitial fluid pressure in tumor tissues; and the drug has a high utilization rate and is more fully absorbed. Alyami et al. (78) conducted a retrospective analysis of the prospective maintenance of the PIPAC database in patients with unresectable gastric cancer peritoneal metastasis. They found that when PIPAC was combined with systemic chemotherapy of cisplatin 7.5 mg/m^2^ and doxorubicin 1.5 mg/m^2^, patients experienced fewer complications and significantly improved survival rates. In a prospective study of patients with peritoneal metastatic colorectal cancer, appendiceal cancer, and small intestine cancer, Gockel et al. (79) found that up to 86% of patients who received repeated PIPAC treatment had reduced or stabilized MA volume, and only one patient showed an increase in MA. The above indicates that PIPAC is a feasible, safe, and well-tolerated method. However, currently, all relevant studies are retrospective and prospective and not included in randomized controlled trials, and due to the relatively novel nature of this technology, its long-term efficacy and safety still need further validation (80).

##### 5.4.2.3 Hyperthermic intraperitoneal chemotherapy (HIPEC)

This is a combination of hyperthermia and chemotherapy. Compared with simple intraperitoneal perfusion, intraperitoneal thermal perfusion has a significant increase in short-term objective response rate. Hyperthermic intraperitoneal chemotherapy is the process of heating an infusion solution containing chemotherapy drugs to a certain temperature (usually 42°C (81), which poses less threat to normal cells and better utilizes the synergistic effect of thermal therapy and chemotherapy) and continuously perfusing the patient’s abdominal and pelvic circulation at a constant temperature for a certain period. This method can produce a synergistic sensitization effect of thermal chemotherapy while utilizing the flushing effect of circulating perfusion, removing cancer cells and small lesions from the body cavity, reducing the risk of residual lesions, and improving the thoroughness of treatment. At present, many clinical studies have confirmed that the effective rate of intraperitoneal thermal perfusion chemotherapy for treating MA is 84% to 100% (82). Heat causes greater damage to cancer cells than normal cells. It can increase the absorption and penetration of chemotherapy drugs by malignant cells by increasing membrane permeability and improving membrane transport. Heat can also cause changes in drug pharmacokinetics and enhance the cytotoxicity of chemotherapy drugs (83). In a study targeting patients with advanced peritoneal disseminated ovarian cancer (84), a total of 36 individuals who developed resistance to systemic chemotherapy were included. It was found that HIPEC could significantly improve survival benefits: The 1-year overall survival rate (OSR) was as high as 65% ± 8%, and the 5-year OSR was 16% ± 7%. Of particular note, the MA of most patients disappeared after three to five sessions of HIPEC, effectively improving their quality of life and significantly reducing associated adverse reactions compared to systemic chemotherapy. Moreover, even after receiving up to 25 HIPEC treatments, some patients still maintained a response to the treatment, demonstrating the sustained effectiveness of this therapy. In addition, for 15 patients with refractory gastric cancer accompanied by peritoneal metastasis (85), the combination therapy strategy of mitomycin C combined with HIPEC and large-scale surgery successfully achieved comprehensive destruction of peritoneal effusion and cancer cells inside and outside the peritoneum, further confirming the potential of HIPEC in MA treatment. Another study on 107 patients with advanced ovarian cancer showed that (86) the combination of the TP chemotherapy regimen and HIPEC significantly enhanced the patients’ immune function. In addition, this study also found that compared to the TP group, the combined HIPEC therapy group had a more significant occurrence of gastrointestinal reactions (*P* = 0.034), suggesting that attention should be paid to and related side effects should be managed when optimizing treatment plans.

##### 5.4.2.4 Foam-based intraperitoneal chemotherapy (FBIC)

In the past, intraperitoneal chemotherapy (IPC) commonly used liquid solutions injected into the abdominal cavity. Recently, an innovative strategy has been proposed to use foam as the drug delivery carrier of IPC. Khosrawipour C (87) and his team were the first to comprehensively explore the practical application of FBIC *in vivo*, including its feasibility, scalability, drug distribution, and penetration depth. Their study treated three experimental pigs with enhanced FBIC doxorubicin based on a bicarbonate carrier system, followed by abdominal CT scans. The results showed that enhanced FBIC doxorubicin successfully covered the entire abdominal area. In addition, the team’s subsequent research further demonstrated the feasibility of FBIC in intraoperative anesthesia assistance and postoperative management and basically verified that the safety and biocompatibility of FBIC reached the ideal level (88), opening up a new, safe, and effective treatment path for intra-abdominal chemotherapy.

At the same time, Schubert and other scholars (89) were also committed to the research of FBIC. They prepared FBIC foam with taurine butyl, hydrogen peroxide, human serum, potassium iodide, doxorubicin/oxaliplatin, and other raw materials and conducted detailed experiments. The results showed that compared with the control group and traditional liquid IPC, FBIC showed higher permeability (approximately 275 ± 87 µm) and stronger cytotoxicity (*P* < 0.005). The volume of the generated foam was approximately 50 times the initial liquid solution, and the foam was stable. Its core temperature can rapidly rise to 47°C in 9 min, ensuring the effective release and distribution of drug uniformity.

In summary, the above research demonstrates the potential of FBIC as a treatment method with broad clinical application prospects and provides new strategies and tools for controlling MA. The chemotherapy approaches in the treatment of MA mentioned above are summarized in **Table 2**.

**Table 2:** Chemotherapy approaches in the management of malignant ascites.

Systemic chemotherapy	Ovarian cancer	CAP (72) (cyclophosphamide, adriamycin, cisplatin)
		paclitaxel combined with carboplatin
	Lymphoma	CHOP (73) (cyclophosphamide, vincristine, adriamycin, prednisone)
	Breast cancer	CAF (74) (cyclophosphamide, adriamycin, 5-fluorouracil)
Intraperitoneal chemotherapy (75)	General intraperitoneal chemotherapy (76)	
	Pressurized intraperitoneal aerosol chemotherapy (PIPAC) (77–80)	
	Hyperthermic intraperitoneal chemotherapy (HIPEC) (81–86)	
	Foam-based intraperitoneal chemotherapy (FBIC) (87–89)	
	Gas-based intraperitoneal hyperthermia (GIH) (90–93)	

### 5.5 Other intraperitoneal administrations

#### 5.5.1 Anti-angiogenic therapy

Since VEGF is a key factor that triggers ascites, injecting VEGF monoclonal antibodies or VEGF inhibitors into the abdominal cavity, such as bevacizumab and ranibizumab, can be used for the treatment of MA and has achieved certain therapeutic effects. In a clinical study conducted by Zhou et al. (94), in addition to receiving cisplatin intraperitoneal infusion therapy, the observation group also received bevacizumab intraperitoneal infusion therapy for MA. In contrast, the control group only received cisplatin intraperitoneal infusion. The results showed that the observation group’s MA volume and average urine volume were significantly improved, and the difference was statistically significant compared to the control group. Chen Peng et al. (95) used sequential intraperitoneal infusion of endostatin (endostatin) combined with docetaxel and intraperitoneal infusion of docetaxel alone to treat MA. They found that the total effective rate of the endostatin combined with the docetaxel group was 96.55%, and the adverse reaction rate was 6.90%. These data were significantly better than those of the docetaxel alone group.

#### 5.5.2 Matrix metalloproteinase inhibitors

MMPs are an enzyme family overexpressed in multiple types of cancer. Intraperitoneal injection of MMP inhibitors can effectively slow down tumor growth, metastatic spread, and the generation of MA (50).

#### 5.5.3 Biological response regulators

Interleukin-2 (IL-2), interferon (96), BCG vaccine (97), tumor necrosis factor-alpha (TNF-α) and streptococcal preparation (OK-432), highly agglutinative staphylococcin (HAS) (98), *Nocardia rubra* cell wall skeleton (99), *Corynebacterium parvum* (100), and somatostatin (101) can be used alone or in combination with other drugs for the treatment of MA. The mechanism by which these modulators work is related to their activation of the immune system, making cancer cells more sensitive to the body’s defense mechanisms or the effects of anticancer drugs. Guo Nannan et al. (102) used a combination of snake venom hemagglutinin and IL-2 to treat MA, and the therapeutic effect was significantly better than that of the snake venom hemagglutinin combined with the cisplatin group while significantly improving the quality of life of patients. Qin Shukui et al. (103) reported the use of recombinant human TNF-α for single-drug intracavitary infusion therapy for MA. In 302 MA patients, the objective response rate reached 46.03%, and the disease control rate was as high as 97.27%. Although biological agents have definite therapeutic effects in treating ascites, some researchers have pointed out that the method of infusing inflammatory biological agents into the abdominal cavity has limited effectiveness and instead increases the risk of abdominal fibrosis. Clinically, this often leads to intestinal obstruction, which directly leads to a sharp decline in patients’ quality of life.

### 5.6 Surgical treatment

#### 5.6.1 Peritoneal venous shunt

A peritoneal venous shunt (PVS) is a tubular system of one-way valves that can open under specific pressure, allowing fluid to flow from the peritoneal cavity to the superior vena cava. PVS not only helps alleviate symptoms but also has significant advantages over other treatment methods in saving electrolytes and proteins, maintaining fluid balance and avoiding repeated puncture surgeries (104). Due to its purpose of relieving symptoms (104), PVS is not suitable for patients with short life expectancy (<30 days). Its complications are quite extensive, including postoperative fever, edema, gastrointestinal bleeding, sepsis, tachycardia, ascites leakage, PVS dysfunction or mechanical occlusion, disseminated intravascular coagulation, acute heart failure, and infection. Tumor cells injected into the central venous system may also cause a large number of early metastases. These factors all limit the application of PVS, and its current clinical use is not widespread.

#### 5.6.2 Transjugular intrahepatic portosystemic shunt

Transjugular intrahepatic portosystemic shunt (TIPS) technology, which establishes a portosystemic shunt in the liver parenchyma between the hepatic vein and portal vein, can significantly reduce portal vein resistance structurally. It has certain therapeutic effects on liver cancer with portal hypertension and refractory ascites and can accelerate the discharge of abdominal fluid. In TIPS surgery, there may be a risk of tumor rupture or bleeding when the puncture needle or shunt passes through the tumor. Hong Dong and his team (105) retrospectively analyzed the clinical data of 13 patients with bleeding and ascites caused by hepatocellular carcinoma and portal hypertension. The results showed that the incidence of MA before and after TIPS treatment was 84.6% (11/13) and 7.7% (1/13), respectively. This indicates that TIPS can effectively control MA, bleeding, etc., which helps patients better adapt to subsequent antitumor treatment and achieve a good quality of life.

#### 5.6.3 Abdominal tumor cell reduction surgery

After abdominal exploration, visible peritoneal tumor tissue is removed and reduced to the greatest extent possible, retaining only residual lesions under the microscope. Subsequently, heated chemotherapy drugs were used for intraperitoneal infusion to maximize the elimination of residual cancer cells in the abdominal cavity. Cell reduction surgery (CRS) is most commonly combined with HIPEC. In a meta-analysis on the application of CRS combined with HIPEC in primary ovarian cancer (106), a significant improvement was reported in the 5-year overall survival (OS) (393 cases, hazard ratio = 0.77; 95% confidence interval 0.67–0.90; *P* = 0.001) and disease-free survival (DFS) (hazard ratio = 0.60; 95%; confidence interval 0.41–0.87; *P* = 0.008) after treatment.

### 5.7 Other treatments

#### 5.7.1 Radionuclides

Released from radioactive isotopes, β radiation can kill malignant tumor cells at short distances. In clinical practice, 32P is the most commonly used. Radionuclides have shown better efficacy in the treatment of MA patients with positive exfoliated cells in ascites, diffuse metastasis to the dry peritoneum, or implantation. According to literature reports (107), the highest effective rate of radioactive nuclides in treating MA can reach 80%, and the resolution time of some ascites can even reach up to 6 months. The main complications include radiation-induced enteritis, intestinal obstruction, and intestinal infarction.

#### 5.7.2 Intraperitoneal injection of catumaxomab

Catumaxomab (108) is a three-functional, non-humanized mouse/rat monoclonal antibody that targets the epithelial cell adhesion molecule (EpCAM) on T cells. In 70%–100% of MA cases, tumor cells commonly express EpCAM (109), and injection of cetuximab can directly reduce the number of circulating tumor cells in the peritoneal cavity, thereby reducing the production of MA. In a phase II/III clinical trial (110), catumaxomab was used to treat symptomatic MA patients with EpCAM-positive tumor recurrence. Compared with puncture alone, the addition of cetuximab significantly prolonged puncture-free survival.

#### 5.7.3 Gas-based intraperitoneal hyperthermia combined with dehydration

Peritoneal metastasis (PM) is a persistent challenge in the treatment of MA. A high-flow gas-based heat shock program that combines hyperthermia and dehydration (dehydrating the peritoneal cavity, which induces peritoneal drying and cancer cell necrosis on the peritoneal surface) has become a new method for treating PM.

Diakun A and other scholars (90) have innovatively explored the potential of gas-based intraperitoneal hyperthermia (GIH) and pointed out that, without causing harmful increases in the body’s core temperature, GIH can be safely implemented within the range of 48°C–50°C by utilizing the low specific heat capacity of gaseous substances (91), effectively inducing a decrease in HT-29 colon cancer cell viability and an increase in cytotoxicity. This study is the first to evaluate the therapeutic potential of *in-vivo* dehydration, hyperthermia, and their combination in animal models (pigs). Using diagnostic laparoscopic techniques, high-flow airflow treatment was applied at specific temperatures (48°C, 49°C, 50°C) to measure cell toxicity and viability at different time intervals. The results showed that both dehydration and hyperthermia had cytotoxicity on HT-29 cells, and the effect of dehydration on cell viability was significantly increased when combined with hyperthermia (*P* < 0.01) (92).

In addition, the study of Khosrawipour C et al. (93) further expanded the boundaries of the combined application of dehydration and chemotherapy under high-temperature conditions. They simulated a high-temperature environment to partially dehydrate HT-29 colon cancer cells, followed by chemotherapy intervention with oxaliplatin or doxorubicin. They found that compared to chemotherapy alone, this combination therapy significantly improved the overall cytotoxicity of colon cancer cells. This discovery not only confirms the superiority of the synergistic effect of high-temperature dehydration and chemotherapy but also provides strong scientific support for the comprehensive treatment strategy of PM.

#### 5.7.4 Nucleic acid treatments

Nucleic acid treatments (111) including the application of messenger RNA (mRNA), small interfering RNA (siRNA), and microRNA (miRNA) work by precisely regulating the expression and silencing of specific genes or gene products closely related to cancer growth and progression, achieving precise targeted therapy for cancer.

##### 5.7.4.1 mRNA therapy

With the continuous advancement of nucleic acid therapy technology, mRNA lipid nanoparticles (LNPs), as a novel carrier, can efficiently deliver mRNA encoding therapeutic follicular proteins into cancer cells after intraperitoneal administration, thereby exerting their therapeutic effects (112). A study on a mouse model of HER2-positive breast cancer revealed the significant potential of mRNA therapy (113). In the study, follistatin (FST) mRNA prevented MA, delayed cancer progression, reduced solid tumor volume, and effectively reduced muscle loss by effectively inhibiting negative muscle regulatory factors. Another study (114) revealed that FST mRNA therapy using lipid nanoparticles as carriers can effectively inhibit the abnormal increase in activator A levels caused by invasive ovarian cancer and its associated cachexia state. In addition, the study further observed that when FST mRNA therapy was combined with the chemotherapy drug cisplatin, the two showed significant synergistic effects, significantly improving the survival rate of experimental mice.

##### 5.7.4.2 siRNA therapy

The mechanism of action of siRNA therapy based on nanoparticles is to precisely deliver siRNA molecules into the cytoplasm of cancer cells. Here, siRNA can specifically target and interfere with the normal function of resistance-related genes, thereby reducing multidrug resistance in gynecological cancers and enhancing the sensitivity of cancer cells to chemotherapy drugs (115). However, it is worth noting that siRNA therapy may promote genes related to fluid accumulation, including VEGF, leptin, and IL-10 (111).

##### 5.7.4.3 miRNA therapy

miRNA is a short non-coding RNA that has become a potential target in the field of gynecological cancer treatment. The formation of malignant ascites is often closely related to the epithelial–mesenchymal transition (EMT) process. During the EMT process, cancer cells gain strong mobility and invasiveness from stromal cells, allowing them to separate from the primary tumor and spread into the peritoneal cavity, ultimately forming MA (116). Recently, a study on an ID8-VEGF mouse ovarian cancer model (117) found that compared to monotherapy, paclitaxel combined with anti-Let-7b miRNA therapy can significantly reduce VEGF levels in peritoneal fluid. Anti-Let-7b miRNA effectively reduces ascites production in mice by precisely regulating specific genes and signaling pathways involved in the EMT process.

In the field of MA treatment, nucleic acid-based cutting-edge therapies have become potential strategies for selectively targeting genes that drive cancer growth and promote the spread of malignant tumors, and are worthy of further exploration. According to the entire text, the mechanism, diagnosis, and treatment methods of MA are summarized in (**Table 3**).

**Table 3:** The mechanism, diagnosis, and treatment methods of malignant ascites.

Production mechanism	Obstruction of lymph and tissue fluid return (3)		
	Neovascularization and increased vascular permeability (4–10)		
	Increased permeability of microvascular walls (11)		
	Reduced plasma colloid osmotic pressure (12, 13)		
	Activation of relevant regulatory mechanisms (14, 15)		
	Chylous fluid leakage (16)		
Diagnosis	Laboratory diagnostic methods	Routine examination of MA (24)	
		Biochemical examination of MA (25–29)	Protein, ferritin, SAAG, FN, LDH, glucose (28), TC, lysozyme, ADA
		Ascites cytological examination (31)	The gold standard for diagnosing MA
		Tumor marker (32–36)	CEA, AFP, CA125, CA19-9
	Physical examination (37–39)	Clinical symptoms, abdominal palpation, percussion	
	Imaging examination	X-ray (35)	
		CT and MRI (40–44)	
		B-ultrasound (45–47)	Most sensitive
	Molecular biology examination	Vascular endothelial growth factor (VEGF) (48)	
		Matrix metalloproteinases (MMPs) (49, 50)	
		Telomerase (51, 52)	
		Transforming growth factors β1 (TGF-β1) (53, 54)	
	Laparoscopic examination combined with peritoneal biopsy (55)		
Treatment	Targeted supportive treatment (58, 59)		
	Diuretic treatment (61–68)	Furosemide (62)	
		Spironolactone (63)	
		Hydrochlorothiazide (63)	
	Abdominal paracentesis	Abdominal puncture drainage (20, 69–71, 118)	
		Indwelling peritoneal catheter for abdominal drainage (68)	
	Chemotherapy	Systemic chemotherapy (72–74)	Refer to systematic chemotherapy guidelines
		Intraperitoneal chemotherapy (73)	General intraperitoneal chemotherapy (76)
			Pressurized intraperitoneal aerosol chemotherapy (PIPAC) (77–80)
			Hyperthermic intraperitoneal chemotherapy (HIPEC) (81–86)
			Foam-based intraperitoneal chemotherapy(FBIC) (87–89)
			Gas-based intraperitoneal hyperthermia (GIH) (90–93)
	Other intraperitoneal administrations	Antiangiogenic therapy	VEGF monoclonal antibody or VEGF inhibitors (94, 95)
		MMP inhibitors (50)	
		Biological response regulators	IL-2, interferon (96), BCG vaccine (97), TNF-α, OK-432, HAS (98), *Nocardia rubra* cell wall skeleton (99), somatostatin (101), *Corynebacterium parvum* (100), etc.
	Surgical treatment	Peritoneal venous shunt (PVS) (104)	
		Transjugular intrahepatic portosystemic shunt (TIPS) (105)	
		Abdominal tumor cell reduction surgery (CRS) (106)	
	Other treatments	Radionuclides (107)	
		Intraperitoneal injection of catumaxomab (108–110)	
		Gas-based intraperitoneal hyperthermia (GIH) combined with dehydration (90–93)	
		Nucleic acid treatments (111–117)	

## 6 Conclusion

The treatment and management of MA is a complex interdisciplinary problem that requires collaborative treatment from multiple disciplines. On the basis of traditional treatment methods, we should actively explore and apply emerging treatment methods to synergistically inhibit the growth and spread of cancer cells, reduce the production of MA, and thus improve the quality of life and survival of cancer patients. The treatment strategy for MA must go beyond simply controlling ascites and focus on the comprehensive control of the tumor itself. The key to fundamentally reducing ascites and improving patient prognosis is to effectively reduce tumor volume and burden through comprehensive treatment methods such as chemotherapy, radiotherapy, and surgery. In addition, for late-stage patients, palliative care and psychological support should also be emphasized to help patients alleviate physical and mental pain and improve their quality of life.

Faced with the current situation of insufficient research evidence and lack of practical guidelines in the field of MA treatment, we should encourage and promote the in-depth development of basic research and clinical practice, especially through high-quality research designs such as large sample, multicenter, randomized controlled trials, etc., to systematically collect and analyze data on the pathogenesis, diagnostic technology innovation, treatment strategy optimization, and prognosis evaluation of MA, providing strong support for the development of scientific and standardized treatment guidelines. In addition, strengthening international scientific research cooperation and academic exchanges is a key path to achieving breakthrough progress in the field of MA treatment. By sharing the latest research results, exchanging valuable treatment experience, and collaborating to overcome technical difficulties, we can effectively narrow the cognitive gap and jointly promote the innovation of MA treatment technology and concepts. In short, the treatment and management of MA is a difficult and complex task that requires us to gather multidisciplinary wisdom, be brave in innovation and exploration, and adhere to the continuous promotion of high-quality research. Only in this way can we tailor a more comprehensive, effective, and humane treatment plan for cancer patients with MA, helping them alleviate their pain and regain hope and dignity in life.
